# Diagnostic Significance of hsa-miR-21-5p, hsa-miR-192-5p, hsa-miR-155-5p, hsa-miR-199a-5p Panel and Ratios in Hepatocellular Carcinoma on Top of Liver Cirrhosis in HCV-Infected Patients

**DOI:** 10.3390/ijms24043157

**Published:** 2023-02-05

**Authors:** Mona A. Eldosoky, Reham Hammad, Asmaa A. Elmadbouly, Reda Badr Aglan, Sherihan G. Abdel-Hamid, Mohamed Alboraie, Donia Ahmed Hassan, Mohamed A. Shaheen, Areej Rushdi, Reem M. Ahmed, Alzahra Abdelbadea, Neamat A. Abdelmageed, Ahmed Elshafei, Elham Ali, Omaima I. Abo-Elkheir, Samy Zaky, Nadia M. Hamdy, Claude Lambert

**Affiliations:** 1 Clinical Pathology Department, Faculty of Medicine (for Girls), Al-Azhar University, Nasr City 11884, Egypt; 2 Hepatology and Gastroenterology Department, National Liver Institute, Menoufia University, Shibin El-Kom 32514, Egypt; 3 Biochemistry Department, Faculty of Pharmacy, Ain Shams University, Cairo 11566, Egypt; 4 Department of Internal Medicine, Al-Azhar University, Cairo 11884, Egypt; 5 Clinical Pathology Department, Faculty of Medicine (for Boys), Al-Azhar University, Cairo 11884, Egypt; 6 Microbiology and Immunology Department, Faculty of Medicine for Girls, Al-Azhar University, Cairo 11884, Egypt; 7 Medical Biochemistry and Molecular Biology, Faculty of Medicine for Girls, Al-Azhar University, Cairo 11884, Egypt; 8 Hepatology, Gastroenterology and Infectious Diseases Department, Faculty of Medicine (for Girls), Al-Azhar University, Cairo 11884, Egypt; 9 Biochemistry and Molecular Biology Department, Faculty of Pharmacy (Boys), Al-Azhar University, Cairo 11884, Egypt; 10 Molecular Biology, Zoology and Entomology Department, Faculty of Science (for Girls), Al-Azhar University, Cairo 11884, Egypt; 11 Community Medicine and Public Health, Faculty of Medicine, Al-Azhar University, Cairo 11884, Egypt; 12 Cytometry Unit, Immunology Laboratory, Saint-Etienne University Hospital, 42100 Saint-Etienne, France

**Keywords:** HCC, hsa-miR-21-5p, hsa-miR-155-5p, hsa-miR-192-5p, hsa-miR-199a-5p, liver cirrhosis, HCV, AFP-negative HCC, bioinformatics/in silico analysis

## Abstract

Early hepatocellular carcinoma (HCC) diagnosis is challenging. Moreover, for patients with alpha-fetoprotein (AFP)-negative HCC, this challenge is augmented. MicroRNAs (miRs) profiles may serve as potential HCC molecular markers. We aimed to assess plasma homo sapiens—(hsa)-miR-21-5p, hsa-miR-155-5p, hsa-miR-192-5p, and hsa-miR-199a-5p—expression levels as a panel of biomarkers for HCC in chronic hepatitis C virus (CHCV) patients with liver cirrhosis (LC), especially AFP-negative HCC cases, as a step toward non-protein coding (nc) RNA precision medicine. Subjects and methods: 79 patients enrolled with CHCV infection with LC, subclassified into an LC group without HCC (n = 40) and LC with HCC (n = 39). Real-time quantitative PCR was used to measure plasma hsa-miR-21-5p, hsa-miR-155-5p, hsa-miR-192-5p, and hsa-miR-199a-5p. Results: Plasma hsa-miR-21-5p and hsa-miR-155-5p demonstrated significant upregulation, while hsa-miR-199a-5p demonstrated significant downregulation in the HCC group (n = 39) when compared to the LC group (n = 40). hsa-miR-21-5p expression was positively correlated with serum AFP, insulin, and insulin resistance (*r* = 0.5, *p* < 0.001, *r* = 0.334, *p* = 0.01, and *r* = 0.303, *p* = 0.02, respectively). According to the ROC curves, for differentiating HCC from LC, combining AFP with each of hsa-miR-21-5p, hsa-miR-155-5p, and miR199a-5p improved the diagnostic sensitivity to 87%, 82%, and 84%, respectively, vs. 69% for AFP alone, with acceptable specificities of 77.5%, 77.5%, and 80%, respectively, and AUC = 0.89, 0.85, and 0.90, respectively vs. 0.85 for AFP alone. hsa-miR-21-5p/hsa-miR-199a-5p and hsa-miR-155-5p/hsa-miR-199a-5p ratios discriminated HCC from LC at AUC = 0.76 and 0.71, respectively, with sensitivities = 94% and 92% and specificities = 48% and 53%, respectively. Upregulation of plasma hsa-miR-21-5p was considered as an independent risk factor for HCC development [OR = 1.198(1.063–1.329), *p* = 0.002]. Conclusions: Combining each of hsa-miR-21-5p, hsa-miR-155-5p, and hsa-miR-199a-5p with AFP made it possible to identify HCC development in the LC patients’ cohort with higher sensitivity than using AFP alone. hsa-miR-21-5p/hsa-miR-199a-5p and hsa-miR-155-5p/hsa-miR-199a-5p ratios are potential HCC molecular markers for AFP-negative HCC patients. hsa-miR-21-5p was linked, clinically and via in silico proof, to insulin metabolism, inflammation, dyslipidemia, and tumorigenesis in the HCC patients’ group as well as for an upregulated independent risk factor for the emergence of HCC from LC in the CHCV patients.

## 1. Introduction

Globally, hepatocellular carcinoma (HCC) has the third-highest fatality rate from a highly spreading disease, and when symptomatically diagnosed, the tumor is in an advanced stage [1]. Serum alpha-fetoprotein (AFP) measurement helps in the detection of HCC. However, one-third of HCC patients are AFP-negative HCC, presenting a difficult obstacle to overcome in the clinical practice setting [2]. AFP-negative HCC cases are defined as those with AFP levels lower than 20 ng/mL despite the presence of pathology-confirmed HCC [3]. Hence, finding a more sensitive and reliable biomarker to forecast HCC development is mandatory. Hence, intensive ongoing research is focusing on identifying potential molecular markers for early HCC detection, disease prediction, and/or prognosis. Tumor epigenetics is one of the potential molecular markers that might characterize HCC [4]. Hence, elaborating on the HCC tumor biology is of great value for HCC patients [5], namely for diagnosis or identifying possible treatment targets. Moreover, epigenetic molecular markers can be easily detected and quantified in peripheral blood liquid biopsy, which is a minimally invasive tool that can identify cancer at an early stage as well as monitor disease progression [6]. Micro-RNAs (miRs) are a group of epigenetic small non-protein coding RNA (ncRNA) with 18–22 nucleotide length [7]. According to miRBase https://www.mirbase.org/ (accessed on 28 October 2022), which is one of the microRNA databases (Release 22.1), the miR count is 38,589 entries. MiRs bind to messenger RNAs (mRNA), sponging them to control their intended protein production [8], a mechanism linked to carcinogenesis for one of the different cancer hallmarks [9]. In addition, miRs profiles may serve as potential HCC diagnostic and/or prognostic molecular markers with non-invasive and sensitive properties [10]. Accordingly, if miRs can detect HCC cases, this idea would be a potential step toward the use of ncRNA. For precision health, especially in AFP-negative HCC conditions, AFP cannot be used as an HCC screening marker. One of the most frequently dysregulated microRNAs in cancer is hsa-miR-21-5p. Tissue hsa-miR-21-5p’s diagnostic and prognostic utility has been proven [5]. Altered liver tissue hsa-miR-21-5p can cause altered lipid metabolism, inflammation, and fibrosis with activation of the intracellular oncogenic signaling pathways phosphatidylinositol 3 kinases (PI3K) to protein kinase B (PKB:Akt), transforming growth factor beta (TGF-ß) to the signal transducers suppressor of mothers against decapentaplegic 2 (SMAD2) and to the transcription factor signal transducer and activator of transcription 3 (STAT3), leading to HCC initiation [11]. However, the diagnostic significance of circulating hsa-miR-21-5p in liver cirrhosis or AFP-negative HCC cases has not yet been studied. Monitoring hsa-miR-155-5p expression levels may be helpful in HCV cases, each of which develops cirrhosis and HCC, as described earlier by Mohamed et al. [12]. Per hsa-miR-155-5p is a regulator of the pro-inflammatory precursor mediators nuclear factor kappa-B cell (NF-KB), epidermal growth factor (EGF), and others, so hsa-miR-155-5p would be connected to both HCC and CHCV infection [13]. On the other hand, hsa-miR-192-5p was found to be downregulated in some tumor tissues [14]. However, hsa-miR-192-5p expression level in HCC tissue related to HBV infection was associated with a faster progression of HCC [15]. Therefore, exploration of the CHCV-associated plasma hsa-miR-192-5p expression levels in HCC remains to be conducted. Huang et al. [16] found that hsa-miR-199a-5p expression was lower in HCC tissues than in nearby non-tumor tissues. Recently, hsa-miR-199a-5p mimics achieved less HCC cell line survival or colony formation via decreasing the expression of hypoxia-induced factor-1 (HIF-1) [17]. Nevertheless, the effect of blood hsa-miR-199a-5p was similar to that of the now-mentioned miRs (hsa-miR-21-5p, hsa-miR-155-5p, and hsa-miR-192-5p) together as a unit/panel; HCC must be explored in Egyptian patients with cirrhosis associated with CHCV infection. Hence, in order to improve the sensitivity of HCC diagnosis, we thought to elucidate the diagnostic utility of circulating plasma hsa-miR-21-5p, hsa-miR-155-5p, hsa-miR-192-5p, and hsa-miR-199a-5p expression levels, given non-invasive molecular markers, as a panel or as ratios, for HCC in CHCV-infected Egyptian patients with liver cirrhosis. Considering the diagnostic utility of these miR groups or ratios for AFP-negative HCC cases is a novel potential outcome of this study.

## 2. Results

### 2.1. Bioinformatics Databases Analysis

Bioinformatics identification of the investigated miRs panel (Table 1) (accessed on 28 April 2022) retrieved from miRDB https://mirdb.org/mirdb/index.html and human ncRNA gene database GeneCaRNA https://www.genecards.org/genecarna as well as the miRPathDB v2.0 https://mpd.bioinf.uni-sb.de/overview.html.

### 2.2. Study Data Analysis

Demographic and biochemical, clinical data snd characteristics analysis of study participants are shown in Table 2. No difference between gender and age, BMI or diabetes status, liver and kideny function tests between the two studied groups.

### 2.3. Pathological Characteristics of the HCC Cases Are Shown in Table 3

Table 3 represents the pathologic characteristics of the studied HCC cases (n = 39) and liver cirrhosis cases (n = 40), revealing that 24 cases of the HCC group had no ascites, while 15 patients had ascites graded (8 patients had minor ascites, 5 patients were moderately ascitic, and 2 patients were markedly ascetic). In the cirrhotic group, 16 patients had no ascites and 24 patients had ascites graded, as 6 patients were minimally ascetic, 16 patients were moderately ascetic, and 2 patients were markedly ascetic. Ascites in both groups were explained by the presence of portal hypertension and hypoalbuminemia. All cirrhotic group patients had no LN enlargement, and their portal veins were patent. –In total, 5 patients of the HCC group had LN enlargement (metastatic), and 11 HCC patients had portal vein thrombosis; 9 of them were totally thrombosed, and 2 patients exhibited partially thrombosed portal vein (malignant invasion of the portal vein). Child–Pugh score, which estimates the severity of liver disease, was calculated for all patients. The HCC group had 24 patients with the least severe liver disease (Child A), 10 patients had a moderately severe liver disease (Child B), and 5 patients had the most severe liver disease (Child C). In the cirrhotic group, 12 patients were Child A, 14 patients were Child B, and 14 patients were Child C. HCC patients were classified according to the Barcelona Clinic Liver Cancer (BCLC) classification based on Child scores, number and size of focal lesions, performance status, vascular invasion, and distant metastases. In total, 12 HCC patients were in the early stage (A), 10 patients were in an intermediate stage (B), 12 patients exhibited an advanced stage (C), and finally, 5 patients were in the terminal stage (D). Fortunately, this classification is highly significant in clarifying the best modality of treatment for each stage and also predicts the expected survival.

**Table 3:** Pathological characteristics of the studied HCC cases (n = 39) and liver cirrhosis cases (n = 40).

Groups, n (%)	HCC, 39 (100%)	Liver Cirrhosis, 40 (100%)	Statistics Test, *p*-Value
**Parameters**	Ascites		*X*^2^ = 7.63, 0.05 *
**No.**	24 (61.5%)	16 (40.0%)	
**Minimal**	8 (20.5%)	6 (15.0%)	
**Moderate**	5 (12.8%)	16 (40.0%)	
**Marked or Massive**	2 (5.1%)	2 (5.0%)	
	**LN involvement**		*X*^2^ = 5.475, 0.019 *
**No.**	34 (87.2%)	40 (100.0%)	
**Yes**	5 (12.8%)	0 (0.0%)	
	**Largest liver mass size**		N.A.
**≤3.00 cm**	8 (20.5%)	----	
**>3.00 cm**	31 (79.5%)	----	
	**Portal vein patency**		N.A.
**Patent**	28 (71.8%)	40 (100.0%)	
**Partially occluded**	2 (5.1%)	0 (0.0%)	
**Thrombosed**	9 (23.1%)	0 (0.0%)	
**Subclassification of liver cirrhosis and HCC if found**			
**Child score**			*X*^2^ = 8.9, 0.012 *
**A = Least severe**	24 (61.5%)	12 (30.0%)	
**B = Moderately severe**	10 (25.6%)	14 (35.0%)	
**C = Most severe**	5 (12.8%)	14 (35.0%)	
**BCLC classification**			N.A.
**A = Early stage**	12 (30.8%)	-	
**B = Intermediate stage**	10 (25.6%)	-	
**C = Advanced stage**	12 (30.8%)	-	
**D = Terminal stage**	5 (12.8%)	-	
**Total**	39 (100%)	-	

Data are median (inter quartile range (1st–3rd quartile)), or as number (%), statistics were computed using SPSS software. * Statistical significance *p*-value < 0.05; NS, non-significant; N.A., not applicable. LC, liver cirrhosis; HCC, hepatocellular carcinoma; LN, lymph node; BCLC, Barcelona Clinic Liver Cancer.

As depicted in Table 4, the HCC group showed significant up-regulation of plasma hsa-miR-21-5p expression compared to the LC group (median = 27.66-fold change vs. 8.61-fold change from average expression, *p* < 0.001). In addition, hsa-miR-155-5p expression was significantly elevated in HCC patients in comparison to the cirrhotic patients (median = 3.18-fold change vs. 1.81-fold change, *p* = 0.001). On the other hand, no significant difference was detected between groups regarding hsa-miR-192-5p expression. However, the HCC group showed significant down-regulation of plasma hsa-miR-199a-5p in comparison to the LC group as well as the control group (*p* < 0.05).

When HCC patients were further sub-classified based on AFP detection (cutoff = 20 ng/mL) into AFP-negative HCC patients (n = 12/39) and AFP-positive HCC patients (n = 27/39), significant downregulation of plasma hsa-miR-21-5p levels in AFP-negative HCC patients (*p* = 0.039) was evident, as presented in Table 5.

### 2.4. Correlation Coefficients

Correlation coefficients between studied miRs and various biomarkers in all cases (n = 79): Table 6 highlights that hsa-miR-21-5p expression is positively correlated with AFP, serum insulin and insulin resistance status (*r* = 0.5, *p* < 0.001, *r* = 0.334, *p* = 0.01 and *r* = 0.303, *p* = 0.02). Furthermore, a positive correlation was observed between AFP and hsa-miR-155-5p levels (*r* = 0.371, *p* = 0.001). In addition, hsa-miR-21-5p was positively correlated with dyslipidemia, manifested as high TC and TAG (*r* = 0.241, *p* = 0.033 and *r* = 0.235, *p* = 0.037, respectively).

### 2.5. ROC Curve Analysi

**Figure 1:**
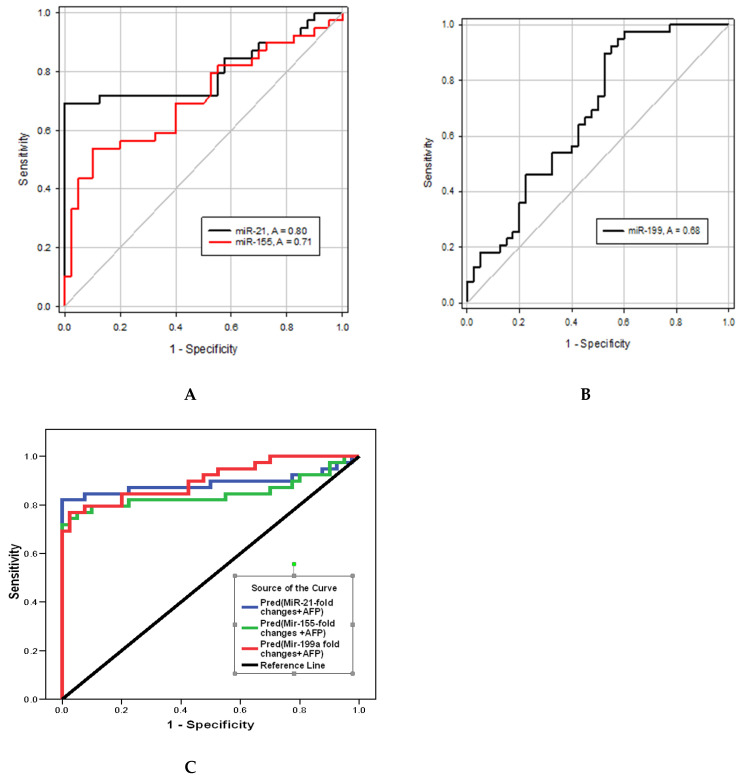
ROC curve for the discriminative ability of the investigated hsa-miRs expression levels to detect CHCV-G4-mediated HCC development from liver cirrhosis; (**A**) hsa-miR-21-5p and hsa-miR-155-5p, (**B**) hsa-miR-199a-5p, (**C**) hsa-miR-21-5p/hsa-miR-199a-5p and hsa-miR-155-5p/hsa-miR-199a-5p.

Table 7 and Figure 1 show that hsa-miR-21-5p upregulation distinguished HCC from LC groups at AUC = 0.8 with 74% sensitivity and 45% specificity at cutoff >7.3-fold increase from average expression, whereas hsa-miR-155-5p distinguished the two groups at AUC = 0.7, 72% sensitivity, and 48% specificity at cutoff >1.8-fold change. hsa-miR-199a-5p distinguished the HCC group from the LC group at AUC = 0.68, 87% sensitivity, and 48% specificity at cutoff <0.45-fold change. Moreover, the hsa-miR-21-5p/hsa-miR-199a-5p ratio could discriminate the HCC from the LC group at AUC = 0.76, 94% sensitivity, and 48% specificity at cutoff >11.45. hsa-miR-155-5p/hsa-miR-199a-5p ratio distinguished the two groups at AUC = 0.71, 92% sensitivity, and 53% specificity at cut-off >2.89.

### 2.6. Logistic Regression Analysis

In order to evaluate if the altered expression panel of hsa-miR-21-5p, hsa-miR-155-5p, hsa-miR-192-5p, and hsa-miR-199a-5p can act as an independent risk factor for HCC progression from liver cirrhosis, a logistic regression analysis was conducted with the adjustment of co-founders (age, BMI, RBS, TAG/HDL), as shown in Table 8. Now, we can say that hsa-miR-21-5p can be considered an independent risk factor for the development of HCC (OR = 1.198 (1.063–1.329, *p* = 0.002)).

### 2.7. Potential Target Genes of Individual miRs and hsa-miRs Panel Predicition In Silico Using the Online Algorithm 

#### 2.7.1. Gene–Gene and Pathways Interactions

**Figure 2:**
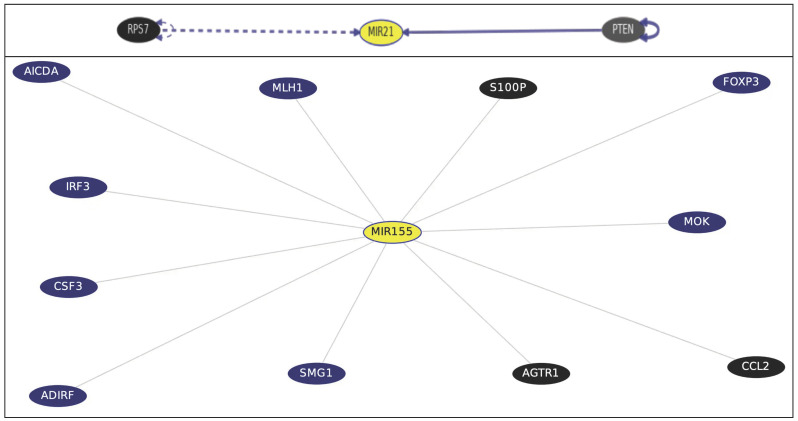
Gene interactions and pathways from curated databases and text-mining for *miR21* and *miR155* genes (in yellow). Black-colored genes are treatment hits by DrugBank. PTEN; phosphatase and tension homolog tumor suppressor; RPS7, ribosomal protein S7; S100P, calcium-binding protein P calmodulin; AGTR1, angiotensin II receptor type 1 G-protein-coupled receptor; CCL2, chemokine (C-C motif) ligand 2.

Gene–Gene Interactions and Pathways from Curated Databases and Text-mining (Figure 2) Via gene-interaction on University of California Santa Cruiz (UCSC) Genomics Institute (accessed on 28 October 2022). The pathways of the studied *miR* genes were completed. *miR21* is directly affected by phosphatase and tensin homolog tumor suppressor (PTEN) (*PTEN* → *miR21*), meaning *PTEN* directly decreases *miR-21* gene as well as the ribosomal protein S7 (RPS7) (http://genome.ucsc.edu/cgi-bin/hgGeneGraph?gene=MIR21&supportLevel=ppi&geneCount=25&geneCount=25&geneAnnot=gnf2&1=OK&lastGene=MIR21). For *miR-155*, top interacting genes that have known inhibitor drugs are calcium-binding protein P calmodulin (S100P), which is targeted by cromoglicic acid (from DrugBank); angiotensin II receptor type 1 G-protein coupled receptor (AGTR1), targeted by the Sartan drug group; chemokine (C-C motif) ligand 2 (CCL2), targeted by Danazol; and mimosine (http://genome.ucsc.edu/cgi-bin/hgGeneGraph?gene=MIR155&supportLevel=text&hideIndirect=on&geneCount=25&geneAnnot=none&1=OK&geneCount=25).

However, hsa-miR-192-5p and hsa-miR-199a-5p genes (*miR192* or *miR199a*) are not present in this current gene interaction database.

#### 2.7.2. hsa-miRs Target(s) Analysis

Through the analysis of hundreds of miRNA–target interactions from high-throughput sequencing experiments, the bioinformatics tool MirTarget was able to create miR database (miRDB), an online library for miR target predictions and functional annotations (https://mirdb.org/custom.html). For hsa-miR-199a-5p target expression analysis, the expression levels of predicted hsa-miR targets retrieved in miRDB (accessed on 28 October 2022) are epidermal growth factor (EGF), mitogen-activated protein 3 kinase 11 (MAP3K11), cell-division-cycle-associated 7-like, and zinc finger proteins.

#### 2.7.3. KEGG Targeted Pathways, Clusters/Heatmap Using DIANA

**Figure 3:**
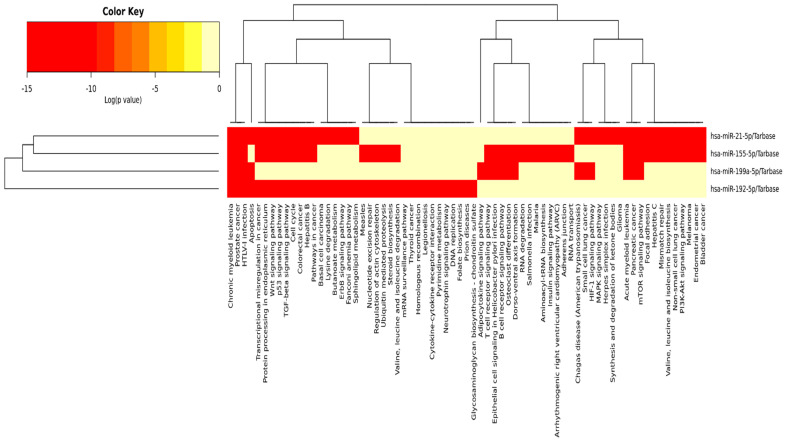
Heatmap showing KEGG pathway intersection/union with all the investigated hsa-miRs; miR cluster dendrogram on the left side of the figure, and KEGG pathways cluster dendrogram in the figure’s upper part. Targeted KEGG pathways, with statistically significant results for all the investigated miRs in red by Tarbase (a posteriori analysis method after an enrichment analysis was performed) (https://dianalab.e-ce.uth.gr/html/universe/index.php?r=mirpath#mirnas=hsa-miR-21-5p;hsa-miR-155-5p;hsa-miR-192-5p;hsa-miR-199a-5p&methods=Tarbase;Tarbase;Tarbase;Tarbase&selection=2). *p* value threshold set at 0.05 and MicroT threshold set at 0.8. Accessed on 18 November 2022.

Mirpath uses reverse search to identify miRs involved in KEGG pathways using the DIANA-TarBase v7.0 method and by searching clusters/heatmap results for KEGG targeted pathways. The investigated miRs are shown in Figure 3 (https://dianalab.e-ce.uth.gr/html/universe/index.php?r=mirpath#mirnas=hsa-miR-21-5p;hsa-miR-155-5p;hsa-miR-192-5p;hsa-miR-199a-5p&methods=Tarbase;Tarbase;Tarbase;Tarbase&selection=2). *p* value threshold is set at 0.05, and microT threshold is set at 0.8. Results are visualized as KEGG pathway unions or as intersected. Intersection of the four KEGG-pathway-investigated miRs are those for chronic myeloid leukemia and several types of cancer. For HCV, two miRs, hsa-miR-21-5p and hsa-miR-155-5p, are involved. However, KEGG pathway unions involving the three miRs—hsa-miR-21-5p, hsa-miR-155-5p, and hsa-miR-192-5p—are those for cancer, cell cycle, HBV, TGF-B, Wnt signaling pathway, and p53 signaling. For mTOR signaling pathway—hsa-miR-21-5p, hsa-miR-155-5p, and hsa-miR-199a-5p—are involved.

#### 2.7.4. Genes That Share Domain with the Investigated MIR Genes

Determined via GenesLikeMe v5.12 https://glm.genecards.org/#results by LifeMap sciences (Barranca Ave, Covina, CA, USA) (accessed on 15 November 2022). The best inferred functional partners found for miR155 are NF-KB, TP53, STAT3, IL6, TNF, miR199a, VEGFA, *miR21,* MAPK8, and TLR4. However, the best inferred functional partners found for *miR192* are as follows, in descending order: IL-6, TP53, INS, PPARG, STAT3, TLR4, VEGFA, BCL2, EGFR, ADIPQ, HIF1A, KRAS, *miR21*, IGF1, and PTEN. Interestingly, the best inferred functional partners found for *miR199A1* were *miR21* and *miR155*.

## 3. Discussion

In the current study, comparison studies revealed that insulin resistance was significantly present in cases of HCC when compared to liver cirrhosis. This finding agrees with many previous studies according to Fujii et al. [18]. The existence of hereditary variables, hepatic fat buildup, changes in energy metabolism, and inflammatory signals originating from immune cells all play a role in how chronic hepatic disease progresses to HCC. Kim et al. [19] stated that diabetes contributes to the biologic processes driving chronic liver diseases to HCC. For lipid metabolism, no significant differences were detected when TC, TAG, and HDL were compared between HCC cases and liver cirrhosis cases. These findings disagree with Sangineto et al.’s [20] findings stating that metabolic reprogramming plays a crucial role in the emergence and spread of cancer. In particular, the role of lipid metabolism in neoplastic cells’ energy production, environmental adaptation, and cell signaling has been studied. This discrepancy could be explained by the multifactorial nature of the pathway driving HCC pathology, which highlights the role of epigenetic factors as miRs in HCC development. AFP expression was significantly upregulated in HCC patients compared to cirrhotic patients. AFP is the most widely used serum biomarker to detect HCC worldwide. This issue agrees with Park et al. [21], who proposed AFP as the best performance biomarker for HCC diagnosis when secreted. However, even if a low-level cutoff is utilized (i.e., 10–20 ng/mL), the sensitivity value of AFP to detect HCC is around 60–70%. In addition, serum AFP levels are normal in 15–30% of HCC cases [22], which justifies the need for the discovery of new HCC markers. In the current study, plasma hsa-miR-21-5p expression was significantly upregulated in HCC patients compared to cirrhotic patients. These findings are in line with the findings of Tian et al. [23], who reported that the exosomal hsa-miR-21-5p is upregulated in HCC due to the acidic microenvironment and that hsa-miR-21-5p activates HIF-1 and HIF-2, promoting the growth and spread of HCC cells. Beyond that, Cao et al. [24] found that hsa-miR-21-5p stimulates HCC development via controlling the expression of tumor suppressor gene PTEN, which prevents tumor cell apoptosis [25]. This fact is witnessed via the bioinformatics analysis in Figure 2, which points to the straight relation of miR-21 to PTEN. Additionally, the HCC group (n = 39) demonstrated significant up-regulation of plasma hsa-miR-155-5p expression level. This event is consistent with the findings of Matsuura et al. [26], in which hsa-miR-155-5p significantly increased angiogenesis in the hypoxic condition generated by HCC. Hsa-miR-155-5p was proved to promote HCC cells’ invasion and migration, being a moderator for epithelial–mesenchymal transition (EMT) [27]. The RefSeq MIR155HG gene is a miR host gene, where the long RNA transcribed from this gene is expressed at high levels in lymphoma and may function as an oncogene, as provided by RefSeq, Dec 2017 http://genome.ucsc.edu/cgi-bin/hgGene?db=hg19&hgg_gene=MIR155. On the other hand, the HCC group demonstrated a significant down-regulation of plasma hsa-miR-199a-5p expression level. This finding was in-line with the finding of a recent publication by Chen et al. [28], who observed that miR-199a-5p was significantly downregulated in tumor samples from HCC patients. According to Liu et al. [29], hsa-miR-199a-5p downregulation plays a crucial role in the growth and the development of HCC, and they added that the protective role of hsa-miR-199a-5p is played through its ability to inhibit HCC cell migration and invasion by targeting the metastasis promoter MAP4K3. Additionally, Huang et al. [16] found that hsa-miR-199a-5p functions as a tumor suppressor in HCC, which explains its frequent downregulation in HCC cases. No significant change was detected for hsa-miR-192-5p when compared between all groups. This finding disagrees with Yin et al. [30], who found that hsa-miR-192-5p loss initiates HCC malignancy. Additionally, Gu et al. [31] reported that has-miR-192-5p silencing by genetic aberrations is a key event in hepatocellular carcinoma development. Discrepancy of findings can be due to different methods of miR detection, as we tested the miRs in patients’ plasma rather than in HCC tissue. Moreover, we thought to investigate miR fold change ratios so as to take advantage of our findings of the two upregulated hsa-miR-21-5p and hsa-miR-155-5p with downregulated hsa-miR-199a-5p. We performed a panel of ratios between studied miRs using hsa-miR-21-5p/hsa-miR-199a-5p ratio and hsa-miR-155-5p/hsa-miR-199a-5p ratio. When compared to the liver cirrhosis group and the control group, the HCC group demonstrated a significant up-regulation of hsa-miR-21-5p/hsa-miR-199a-5p ratio and hsa-miR-155-5p/hsa-miR-199a-5p ratio. Comparing miR expression levels and ratios in subclassified groups of HCC study participants according to AFP positivity revealed only a significant up-regulation of hsa-miR-121-5p expression level in AFP positive HCC cases. When detectable, AFP was found to be positively correlated with both hsa-miR-21-5p and hsa-miR-155-5p levels, pointing to the importance of both miRs as molecular markers in the context of HCC diagnosis [32] being related, as a panel, in part to HCC development and progression. No significant difference in hsa-miR-192-5p levels was detected between the HCC group and the other studied groups. However, Fründt et al. [14] claimed hsa-miR-192-5p might distinguish patients with HCC and those who have LC from the healthy subjects, being downregulated in tumor tissues and thought to have an anti-cancer effect. Yin et al. [30] found that hsa-miR-192-5p has an anti-HCC effect, with the ability to induce HCC cell apoptosis and autophagy via the axis hsa-miR-192-5p/CYR61/Akt signaling pathway. On the other hand, our study showed significant downregulation in plasma hsa-miR-199a-5p expression level in the HCC group only. Lou et al. [33] studied the relationship between hsa-miR-199a-5p and the X-box binding protein 1 (XBP1) and cyclin D axes. They reported hsa-miR-199a-5p to be decreased in HCC tissue, resulting in an increased expression of XBP1 and cyclin D, impacting the cell cycle regulation, suggesting that hsa-miR-199a-5p has an antitumor effect. Moreover, the current bioinformatics analysis for “hsa-miR-199a-5p target expression analysis” retrieved in miRDB, is EGF, MAP3K11, and zinc finger proteins, which are all related to tumorigenesis, thus, hsa-miR-199a-5p would have an antitumor effect a downregulation in its plasma ex-pression level will affect its target protein expression (involved in tumor development). That is why its expression level decrease is considered as a potential good HCC diagnostic molecular marker. Correlation studies in all patients (n = 79) revealed that plasma hsa-miR-21-5p, expression level was positively correlated with serum insulin and the presence of insulin resistance and dyslipidemia (increased TC and TAG). Lin et al. [34] suggested that altered plasma miRs might reflect liver lipid metabolism and said that hepatic miR expression contributes to the development of insulin resistance. This response is per both hsa-miR-21-5p and hsa-miR-155-5p would control the expression of genes involved in hepatic TAG and cholesterol metabolism, evidenced by silencing hepatic miR21 genes, where reduced hepatic inflammation and enhanced fibrosis were achieved [35,36]. This was also evidenced by our bioinformatics analysis in Figure 2 showing the KEGG pathways’ heatmap, in which the investigated miRs are involved in the inflammatory processes, diseases, adipogenesis, and fibrotic diseases. At an AUC = 0.85, AFP was able to distinguish between the HCC and LC groups with only 69% SN and 100% SP (cutoff >23.3). Despite the high SP, the low SN threatens AFP usefulness as the sole HCC screening biomarker. Moreover, AFP would be sometimes high in liver cirrhosis. AFP measurements, if combined with ultrasound for HCC screening, offer additional detection to 6%–8% of cases not previously identified by Ultrasound alone [37].

Blood hsa-miRs are of interest for the research of illness prognosis and the detection of cancer due to their great stability in blood and other liquid biopsy samples [38]. In the current study, combining each of the investigated miRs individually to AFP ROC curve analysis yielded an improved AUC each time compared to either alone. Combination of hsa-miR-21-5p or hsa-miR-155-5p or hsa-miR-199a-5p with AFP yielded an improved AUC compared to AFP alone (0.89, 0.85, and 0.90, respectively, vs. 0.85 for AFP alone). Additionally, an improved sensitivities (87%, 82%, and 84%, respectively, vs. 69% SN for AFP alone) with accepted specificities (77.5%, 77.5%, and 80%, respectively) were obtained from such combinations. During the ROC curve analysis, we decided to increase AFP cutoff to 23.3 ng/mL to ensure the inclusion of AFP-negative HCC cases (12/39) [32]. However, Malik et al. [38] stated that hsa-miR-21-5p alone or in combination with AFP did not improve the diagnostic performance of the protein biomarkers. Moreover, we thought to investigate miRs’ fold change ratios. hsa-miR-21-5p/hsa-miR-199a-5p ratio would detect early HCC development at cutoff >11.45, while hsa-miR-155-5p/hsa-miR-199a-5p ratio distinguished the two groups at cut-off >2.89. Both ratios provided a higher SN for early HCC detection, reaching 95% and 92%, respectively. However, SPs did not improve much. Using these suggested ratio cutoffs revealed a 100% detection rate of HCC in AFP-negative HCC patients (12/39) if using hsa-miR-21-5p/hsa-miR-199a-5p or a 91.6% detection rate of HCC in AFP-negative HCC cases if using hsa-miR-155-5p/hsa-miR-199a-5p ratio as screening molecular markers, which was confirmed with CT scan. This point addresses one of our initial objectives in the current research well: to find sensitive detection biomarkers for HCC development in AFP-negative HCC patients. Logistic regression was performed to ensure the utility of using hsa-miR-21-5p, hsa-miR-155-5p, hsa-miR-192-5p, and hsa-miR199a-5p expression level fold change, as panels or ratios, as predictors of CHCV-mediated HCC development from liver cirrhosis after adjustment for confounders (age, BMI, blood sugar, lipids). Logistic regression revealed that hsa-miR-21-5p (*p* = 0.002, OR = 1.18, 95% CI 1.063–1.329) is a significant predictor of CHCV-mediated HCC development from liver cirrhosis. This fact agrees with Sorop et al. [39] who identified hsa-miR-21-5p using a logistic regression equation as a predictor for HCC diagnosis. Additionally, age of patients was a significant predictor of CHCV-mediated HCC development from liver cirrhosis (*p* = 0.027, OR = 1.132, 95% CI 1.014–1.263). Via bioinformatics analysis using DIANA TOOLS mirPath for multiple microRNA analysis, the web-based miR pathway analysis application is now compiling the pivotal role of miRs. hsa-miR-21-5p, hsa-miR-155-5p, hsa-miR-192-5p, and hsa-miR-199a-5p panels from KEGG pathway intersection of the four investigated miRs for cancer are shown in Figure 3. Hence, during HCC development and progression from CHCV-G4 infection, cell cycle and signaling pathways, apoptosis, TGF-B, Wnt signaling, and p53 signaling and mTOR signaling pathways—“all miRs”—are involved as clusters. Moreover, via GenesLikeMe RELATED GENES, inferred functional partners for *miR155* gene are those involved in the tumorigenesis process: NF-kB, TP53, STAT3, IL-6, TNF, VEGFA, *miR21*, MAPK8, and TLR4. Moreover, the following genes—IL-6, TP53, INS, PPARG, STAT3, TLR4, VEGFA, BCL2, EGFR, ADIPQ, HIF1A, KRAS, *miR21*, IGF1, and PTEN—share a domain with *miR192*. Interestingly, the *miR199A1* gene shares a domain with both the *miR21* and *miR155* genes, supporting the miR cluster dendrogram on the left side of Figure 3, confirming the use of ncRNA panel as a step toward precision health. DrugBank http://genome.ucsc.edu/cgi-bin/hgGeneGraph?gene=MIR155&supportLevel=text&hideIndirect=on&geneCount=25&geneAnnot=none&1=OK&geneCount=25 targeted *miR155* top interacting genes included the anti-inflammatory drugs for S100P—or Sartans—or targeted the synthetic steroid derivatives to inhibit chemokines and drugs that will arrest the cell cycle in the G(1) phase before entry into the S phase. All these drugs will affect *miR155* and *miR21*, and therefore, the investigated panel step-wise as cluster.

**Limitations.** The current study did not include the predictive survival role of the investigated miRs panel—hsa-miR-21-5p, hsa-miR-155-5p, hsa-miR-192-5p, or hsa-miR-199a-5p—in patients with CHCV-G4 linked to HCC (a prospective study to be).

**Strengths related to the current research.** As far as we know, this study is the first to describe the diagnostic utility of hsa-miR-21-5p/hsa-miR-199a-5p and hsa-miR-155-5p/hsa-miR-199a-5p ratios in combination with AFP for an enhanced early diagnosis of clinical CHCV-G4-related HCC and liver cirrhosis. These ratios—hsa-miR-21-5p/hsa-miR-199a-5p and hsa-miR-155-5p/hsa-miR-199a-5p—showed great diagnostic utility in AFP-negative HCC cases.

**Recommendations.** Considering hsa-miR-21-5p and/or hsa-miR-155-5p as potential precision therapeutic target(s) for CHCV-G4-related HCC and liver cirrhosis treatment. This will consider sub-classification examination and testing repurposing drugs (potentially targets to be obtained via miRDB), relying on targeting *miR21* and *miR155* genes, and proofing the mechanism experimentally.

**Sustainability Plan.** Blocking—hsa-miR-21-5p, hsa-miR-155-5p, hsa-miR-192-5p, and hsa-miR-199a-5p—target genes based on the findings from gene–gene interaction networks and KEGG curated databases pathways and text-mining, as potential treatment options based on ncRNA, a step toward precision health.

## 4. Patients and Methods

### 4.1. miRs Selection

Based on literature search, some miRs were examined by our research group and based on bioinformatics identification of the investigated miR panel, retrieved from miRDB [40] (https://mirdb.org/mirdb/index.html) and the human ncRNA gene database, GeneCaRNA [41] (https://www.genecards.org/genecarna; Version 5.13, updated 9 November 2022) as well as the miRTarget [42] Link 2.0 (Released 11 December 2020) (https://ccb-compute.cs.uni-saarland.de/mirtargetlink2) (miRPathDB v2.0 https://mpd.bioinf.uni-sb.de/overview.html).

### 4.2. Study Design

Cross-sectional, controlled, retrospective study.

### 4.3. Sample Size and the Study Power

Based on the previous study by Hammad et al. [43], sample size estimation was performed using the G power* sample size online calculator (https://riskcalc.org/samplesize/#) depending on a two-sided significance level of 0.05 and power (1-beta) of 0.95.

### 4.4. Study Participants

This study enrolled 79 Egyptian patients with chronic hepatitis C virus (CHCV) genotype 4 (G4) (serology confirmed) infection with liver cirrhosis (LC) divided into Group 1—LC patients with early HCC (n = 39)—and Group 2—LC without HCC (n = 40). Patients were recruited from the National Liver Institute, Menoufia University and Al-Zahraa University Hospital. Patient inclusion criteria: imaging criteria in accordance with the recent published recommendations’ guidelines [44] were used to confirm HCC diagnosis at the Pathology Unit. A blind abdominal computed tomography (CT) scan was performed using Siemens 128, Germany, using the following logged information: ascites severity, presence of lymph node (LN) enlargement, cirrhosis or growth pattern, and portal vein (PV) patency. The Child–Pugh score was used to classify patients with cirrhosis [45]. The Barcelona Clinic Liver Cancer (BCLC) classification system was used to staging HCC patients [46] into 0 = very early stage, A = early stage, B = intermediate stage, C = advanced stage, and D = terminal stage. Patient exclusion criteria: patients with a history of alcoholism or autoimmune disease; acute or chronic HBV (as determined by serology); HCC not mediated by CHCV; and patients who were undergoing any type of radiation or chemotherapy for a malignancy other than HCC.

### 4.5. Patients’ Data

Demographic data, including age, gender, and patients’ full histories, were retrieved from the hospital medical records. All patients were asked about family cancer history for recording as well as general clinical examination. For calculating body mass index (BMI) (in kg/m^2^), calculation was performed according to (https://www.nhlbi.nih.gov/health/educational/lose_wt/BMI/bmicalc.htm), with normal weight = 18.5–24.9 kg/m^2^, overweight = 25–29.9 kg/m^2^, obesity = BMI of 30 kg/m^2^.

### 4.6. Blood Sampling

Peripheral venous blood (6 mL) was withdrawn from each participant under strict sterile conditions following standard biosecurity and international safety procedures. Fresh 1 mL of each blood sample was placed into an EDTA tube for CBC. Two mLs blood placed in another EDTA tube centrifuged for 10 min at 1900× *g*, after which the plasma was carefully withdrawn and centrifuged again for 10 min at 16,000× *g* at 4 °C to remove additional cellular nucleic acids attached to cell debris. The supernatant was then transferred to microcentrifuge vials and stored at −80 °C for RNA extraction and quantitative real-time PCR (qRT-PCR) for hsa-miR-21-5p, hsa-miR-155-5p, hsa-miR-192-5p, and hsa-miR-199a-5p. The rest of the blood (3 mL) was transferred into a polymer serum gel separator tube with a clot activator (Kremsmünster, Upper Austria, Greiner Bio-One GmbH, Australia) and left for 15 min at room temperature (24 °C) to clot, followed by 10 min of centrifugation at 10,000× *g* at 4 °C. Sera obtained were aliquoted into Eppendorf tubes and stored at −80 °C until biochemical assessment.

#### 4.6.1. RNA Extraction and qRT-PCR

Plasma miRs were extracted from 200 µL plasma using an miRNeasy commercial kit (Cat. NO. 217004, Qiagen, Germany) according to the manufacturer’s protocol. Purity of the extracted RNA was tested spectrophotometrically at 260/280 nm NanoDrop 2000, (Thermo Fisher Scientific, Altrincham, Cheshire, UK). Synthesis of complementary DNA (cDNA) was carried out using a miRCURY LNA RT Kit (Cat. No. 339340, Qiagen, GmbH, Germany) according to the manufacturer’s instructions. hsa-miR-21-5p, hsa-miR-155-5p, hsa-miR-192-5p, and hsa-miR-199a-5p expression was determined using a miRCURY LNA SYBR^®^ Green PCR Kit (Cat. No. 339345, Qiagen, Germany), following the manufacturer’s protocol, using a RT-PCR Quant Studio 5 system (Applied Biosystems, Waltham, MA, USA). The levels of miRs were normalized using a reference internal housekeeping endogenous control, miR SNORD68. qRT-PCR analyses of the miRs were carried out in triplicate.

The miR-21-5p forward primer sequence (5′-3′) is 5′-ACG TGT TAG CTT ATC AGA CTG-3′, 5′-CCG TTA ATG CTA ATC GTG-3′ for miR-155-5p, 5′-CTG ACC TAT GAA TTG ACA GCC GT-3′ for miR-192-5p, and 5′-GGG CCC AGT GTT CAG ACT AC-3′ for miR-199a-5p. However, the SNORD68 forward primer sequence was 5′-ATC ACT GTA AAA CCG TTC CA-3′.

The qRT-PCR cycling conditions were as follows: 95 °C for two min, then 40 cycles, each of 10 s at 95 °C, 60 s at 56 °C, and 30 s at 70 °C. The delta cycle threshold (Ct) was calculated by subtracting the Ct value of SNORD68 from the Ct values of the target miRs in all samples. Fold changes were calculated using 2-ΔΔCt for relative quantification.

#### 4.6.2. Laboratory Testing

A fully automated hematology analyzer (KX21N, Sysmex corporation, Wakinohama-Kaigandori, Chuo-Ku, and Kobe, Japan) was used to perform a complete blood count (CBC) using fresh EDTA blood samples, in accordance with the manufacturer’s recommendations. INR coagulation assays were performed using an automated coagulation analyzer (Diagnostica Stago STA Compact® and Stago STA Compact, Asnières sur Seine Cedex, France). Routine biochemical tests were conducted using a chemistry autoanalyzer device (Cobas Integra 400 Plus, Roche Diagnostics, GmbH, Mannheim, Germany), following the manufacturer’s instructions. Biochemical analysis included blood albumin, aspartate aminotransferase (AST), alanine aminotransferase (ALT), bilirubin, direct bilirubin, alkaline phosphatase (ALP), gamma GT (GGT), total cholesterol (TC), triacylglycerol (TAG), and high-density lipoprotein (HDL). The electro-chemiluminescence immunoassay (ECLIA) was used to measure the serum alpha-fetoprotein (AFP) using a Cobas 6000 e601 module (Roche Diagnostics, Germany). Insulin was detected in test samples by an enzyme-linked immunosorbent test (ELISA) in solid phase using a HyPrep automated ELISA system with plate reader (Hyperion Inc., Miami, FL, USA), where the color intensity formed is related to its insulin concentration. The normal adult insulin range level is 0–25 mU/L.

#### 4.6.3. Ratios: Indices

Platelets-to-lymphocytes ratio (PLR) is a biomarker for systemic inflammation and is considered indicative to immune-related responses. For CHCV infection, PLR was considered superior to NLR and was used to correlate with disease severity in HCC cases [47].

TAG/HDL-C ratio with a cutoff value of more than the apparently healthy control group is set diagnostic for insulin resistance (IR) [48].

Insulin resistance is considered positive in obese, diabetic, and dyslipidemic patients and in those with insulin levels of 18 mU/mL or more after glucose/meal with disturbed PLR [49,50].

### 4.7. Target Genes, Targeted Pathways Clusters/Heatmap of miRs Predicted In Silico Using Online Algorithms

Genome Browser for target genes using University of California Santa Cruiz (UCSC) [51] Genomics institute (http://genome.ucsc.edu/index.html) and Targeted Pathways Clusters/Heatmap prediction algorithms using DIANA TOOLS [52] Mirpath using reverse search to identify miRs involved in KEGG Pathways; microT v4 and microT-CDS or DIANA-TarBase v7.0 and LncBase (https://dianalab.e-ce.uth.gr/html/universe/index.php?r=mirpath/reverse).

KEGG-targeted pathways [53] (Release 104.1, 1 November 2022, https://www.kegg.jp/kegg/kegg2.html) and clusters/heatmaps of the investigated miRs. Finally, genes sharing domains with the studied miR genes were identified using GenesLikeMe [54] v5.12 (https://glm.genecards.org/#results).

### 4.8. Statistical Analysis

Data collected were coded and analyzed using the Statistical Package for Social Science software (SPSS, Version 17, Chicago, IL, USA). Qualitative data are presented as frequencies (n) and percentages (%).

Data were tested for normality using a Shapiro–Wilk calculator (https://www.statskingdom.com/shapiro-wilk-test-calculator.htmL).

Normally, distributed, variables are presented as mean + S.D. and analyzed using two samples’ independent Students’ *t*-tests for comparison. For non-normally distributed variables, data are presented as median (interquartile range as 1st–3rd quartiles or 25th–75th quartiles), then Mann–Whitney (U) was conducted to compare between any two independent groups. Student’s t-test and the Chi-square χ^2^ test were used to compare quantitative and qualitative normally distributed variables between the patients and control groups, respectively. Spearman’s rho correlation test was used to assess the association between quantitative non-parametric variables. Receiver operating characteristic (ROC) curve was performed to detect the best cutoff, sensitivities (SNs), and specificities (SPs) with a calculated area under the curve (AUC) range from 0 to 1. The higher the AUC, the better the parameter in classifying the outcomes correctly. ROC curve analysis was used to determine the discriminative potential of the studied miRs to differentiate HCC cases from liver cirrhosis cases. A logistic regression analysis was performed to determine the independent factors association of the altered expression of the studied miRs with HCC progression. The level of significance was set at *p*-value < 0.05, confidence level or interval (C.II) as 95% and 5%, respectively.

## 5. Conclusions

Blood hsa-miR-21-5p and hsa-miR-155-5p demonstrated significant upregulation, while hsa-miR-199a-5p was significantly downregulated in the HCC CHCV-G4-infection-related group (n = 39) when compared to the LC group (n = 40) with no HCC.

Combining each of hsa-miR-21-5p, hsa-miR-155-5p, and hsa-miR-199a-5p to AFP as HCC diagnostic markers yielded an improved SN compared to using AFP alone.

Regarding AFP-negative HCC cases, hsa-miR-21-5p/hsa-miR-199a-5p and has-miR-155-5p/hsa-miR-199a-5p ratios can be used to better identify HCC development in LC patients with CHCV-G4 infection, with higher sensitivities. hsa-miR-21-5p plays a role in lipid and insulin metabolism in HCC associated with CHCV-G4 infection cases. hsa-miR-21-5p upregulation is an independent risk factor for the emergence of HCC from liver cirrhosis in the CHCV-G4 patient cohort.

In the current study, hsa-miR-192-5p was not shown to have any clinical significance per HCC development in LC patients.

These findings might encourage the use of the aforementioned epigenetic ncRNA markers in panels or ratios as prospective blood-based molecular markers of benefit, during liver cirrhosis early identification and/or CHCV-G4 infection follow-up, for possible HCC development as well as for ensuring all AFP-negative HCC cases’ identification.

## Figures and Tables

**Table 1:** Investigated hsa-miR information retrieved from different micro-RNA databases.

Mature miR	hsa-miR-21-5p	hsa-miR-155-5p	hsa-miR-192-5p	hsa-miR-199a-5p
**Sequence (5′-3′)**	uagcuuaucagacugauguuga	uuaaugcuaaucgugauagggguu	cugaccuaugaauugacagcc	cccaguguucagacuaccuguuc
**Length**	22	24	21	23
**miRBase ID**	MIMAT0000076	MIMAT0000646	MIMAT0000222	MIMAT0000231
**Similar miRNAs**	hsa-miR-590-5p	-	hsa-miR-215-5p	hsa-miR-199b-5p
**Clustered miRNAs ^#^**	-	-	hsa-miR-194-2 hsa-miR-6750	hsa-miR-3120 has-miR-214
**Genomic location**	chr17:59841266-59841337 (+)	chr21:25573980-25574044 (+)	chr11:64891137-64891246 (-)	chr1:172144535-172144644 chr19:10817426-10817496 (-)

Accessed on 28 April 2022, retrieved from miRDB https://mirdb.org/mirdb/index.html and human ncRNA gene database GeneCaRNA https://www.genecards.org/genecarna as well as the miRPathDB v2.0 https://mpd.bioinf.uni-sb.de/overview.html. ^#^ clustered miRNAs are within 10kb in genome.

**Table 2:** Demographic and clinical data/characteristics (unit) of the HCC group (n = 39), liver cirrhosis group (n = 40) participants compared to each other.

	Group (n)		
Data/Characteristics (Unit)	HCC (39)	LC (40)	*p* Value
**Gender (M/F)**	27/12	28/12	NS
**Age (years)**	61.0 (56.0–67.0)	58.5 (54.25–65.0)	NS
**BMI (Kg/m^2^)**	29.0 (27.0–31.0)	29.9 (27.55–33.2)	NS
**D.M (Yes/No)**	15/24	24/16	NS
**s. Insulin (mIU/L)**	25.0 (15.7–42.5)	13.5 (5.98–20.37)	0.001 *
**Insulin resistance (Yes/No)**	28/11	25/15	0.001 *
**AST (U/L)**	77.0 (62–105.0)	72.0 (62.0–78.0)	NS
**ALT (U/L)**	51.0 (42.0–65.0)	57.0 (50.0–63.7)	NS
**Total Bilirubin (mg/dl)**	1.2 (0.9–2.0)	1.5 (1.0–3.07)	NS
**Direct Bilirubin (mg/dl)**	0.70 (0.40–1.2)	0.8 (0.4–1.95)	NS
**ALP (U/L)**	110 (82.0–155.0)	120 (99.8–132.8)	NS
**GGT (U/L)**	60 (53.0–77.0)	67 (55.3–83.5)	NS
**TC (mg/dl)**	162 (122.0–220)	147 (112-181)	NS
**TAG (mg/dl)**	133 (94.0–193.0)	115 (76.8–147)	NS
**HDL-C (mg/dl)**	34 (26.0–40.0)	36.5 (30.5–41.75)	NS
**TAG/HDL-C ratio**	4.1 (2.6–6.7)	3.3 (2.34–4.59)	NS
**INR**	1.2 (1.1–1.37)	1.5 (1.30–2.06)	<0.001 *

Data are median (inter quartile range (1st–3rd quartile)), statistics were computed using SPSS software, Mann–Whitney test was used (for non-parametric data). *p* indicates comparison between HCC and liver cirrhosis groups; * statistical significance *p*-value < 0.05; NS, non-significant. ALT, alanine aminotransferase; AST, aspartate aminotransferase; AFP, alpha fetoprotein; BMI, body mass index; DM, diabetes mellitus; HCC, hepatocellular carcinoma; HDL, high-density lipoprotein; GGT, gamma glutamyl transferase; LC, liver cirrhosis; s. albumin, serum albumin; s. insulin, serum insulin, TC, total cholesterol; TAG, triacylglycerol.

**Table 4:** Study participants’ hsa-miRs in HCC group (n = 39), liver cirrhosis group (n = 40) compared to each other.

	Group, n		
Parameter (Unit)	HCC, 39	LC, 40	*p* Value
**AFP (ng/mL)**	80 (13–305)	7.4 (4.5–10.37)	<0.001 *
**hsa-miR-21-5p-fold changes**	27.6 (6.9–69.5)	8.6 (3.9–11.3)	<0.001 *
**hsa-miR-155-5p-fold changes**	3.1 (1.7–8.12)	1.8 (0.76–2.2)	0.001 *
**hsa-miR-192-5p-fold changes**	0.90 (0.4–1.52)	1.5 (0.55–5.04)	NS
**hsa-miR-199a-5p fold changes**	0.16 (0.04–0.4)	0.37 (0.08–5.64)	0.046 *
**hsa-miR-21-5p/hsa-miR-199a-5p**	85.6 (27-2759.1)	20.3 (1.1–66.7)	<0.001 *
**hsa-miR-155-5p/hsa-miR-199a-5p**	16.5 (4.4-119.4)	2.7 (0.48–19.1)	0.002 *

Data are median (interquartile range (1st–3rd quartile)), statistics were computed using SPSS software, Mann–Whitney test was used (non-parametric data). *p* indicates comparison between HCC and liver cirrhosis groups. * statistical significance *p*-value <0.05; NS, non-significant. AFP, alpha fetoprotein; HCC, hepatocellular carcinoma; LC, liver cirrhosis.

**Table 5:** Investigated hsa-miRs expression level in HCC study participants’ (n = 39) according to AFP-positivity or negativity.

	AFP -/+ HCC, n		
Characteristics (unit)	AFP-ve HCC, 12	AFP + ve HCC, 27	*p* Value
**hsa-miR-21-5p-fold changes** Min–Max median (inter-quartiles)	1.92–69.5 14.8 (3.0–34.8)	3.29–407.31 32.6 (21.3–116.9)	0.015 *
**hsa-miR-155-5p-fold changes** Min–Max median (inter-quartiles)	0.17–247.3 1.7 (0.46–6.6)	0.80–324.03 3.8 (1.9–11.2)	NS
**hsa-miR-192-5p-fold changes** Min–Max median (inter-quartiles)	0.14–23.2 0.97 (0.45–3.5)	0.09–3.86 0.90 (0.36–1.4)	NS
**hsa-miR-199a-5p fold changes** Min–Max median (inter-quartiles)	0.01–2.38 0.16 (0.035–0.38)	0.00–5.39 0.23 (0.04 –0.63)	NS
**hsa-miR-21-5p/hsa-miR-199a-5p** Min–Max median (inter-quartiles)	12.13–8364.1 52.7 (16.8–311.3)	1.06–121,449.75 87.4 (32.2–3468.3)	NS
**hsa-miR-155-5p/hsa-miR-199a-5p** Min–Max median (inter-quartiles)	2.7–29,737.5 12.3 (3.8–29.9)	0.15–41,764.80 17.4 (6.9–207.9)	NS

Data are median (inter quartile range (1st–3rd quartile)), statistics were computed using SPSS software. * Statistical significance *p*-value < 0.05; NS, non-significant for comparison of AFP–HCC and AFP+HCC sub-classification. HCC, hepatocellular carcinoma; LC, liver cirrhosis; AFP, alpha fetoprotein.

**Table 6:** Spearman’s correlation coefficient (*r*) among the investigated hsa-miRs’ expression levels’ fold changes in all CHCV-G4 patients (liver cirrhosis patients post-HCV) (n = 79).

	CHCV-G4 Patients (n = 79) miRs-Fold Change							
	hsa-miR-21-5p		hsa-miR-155-5p		hsa-miR-192-5p		hsa-miR-199a-5p	
Data/Characteristics (Unit)	*r*	*p*-Value	*r*	*p*-Value	*r*	*p*-Value	*r*	*p*-Value
**Age (Years)**	0.004	NS	−0.004	NS	−0.060	NS	−0.054	NS
**BMI (kg/m^2^)**	0.103	NS	0.059	NS	−0.124	NS	**−0.222**	0.050 *
**s. Insulin (mIU/L)**	**0.334**	0.01 *	0.203	NS	−0.001	NS	−0.125	NS
**AFP (ng/mL)**	**0.534**	<0.001 *	**0.371**	0.001 *	−0.092	NS	−0.023	NS
**AST (U/L)**	0.104	NS	−0.001	NS	0.070	NS	0.03	NS
**ALT (U/L)**	−0.172	NS	−0.138	NS	0.114	NS	−0.068	NS
**ALP (U/L)**	**−0.263**	0.019 *	−0.163	NS	−0.043	NS	0.069	NS
**GGT (U/L)**	−0.144	NS	−0.120	NS	0.106	NS	0.145	NS
**TAG (mg/dL)**	**0.241**	0.033 *	0.180	NS	−0.144	NS	0.133	NS
**TC (mg/dL)**	**0.235**	0.037 *	0.120	NS	**−0.230**	0.042 *	0.075	NS
**HDL-C (mg/dL)**	−0.110	NS	−0.064	NS	−0.062	NS	−0.064	NS
**TAG/HDL-C**	0.195	NS	0.115	NS	−0.085	NS	0.111	NS
**PLR**	−0.045	NS	0.137	NS	−0.119	NS	**0.298**	0.008 *
**Number of liver masses**	−0.027	NS	−0.038	NS	0.010	NS	−0.018	NS
**Insulin resistance**	**0.303**	0.02 *	0.103	NS	0.140	NS	−0.090	NS

Spearman correlation coefficient (*r*) was calculated using SPSS software. * Significant correlation at *p* < 0.05 level (2-tailed); NS, non-significant; LC, liver cirrhosis; ALT, alanine aminotransferase; AST, aspartate aminotransferase; AFP, alpha-fetoprotein; BMI, body mass index; DM, diabetes mellitus; HCC, hepatocellular carcinoma; HDL, high-density lipoprotein; GGT, gamma-glutamyl transferase; LC, liver cirrhosis; PLR, platelet lymphocyte ratio; NLR, neutrophil–lymphocyte ratio; s. insulin, serum insulin; TC, total cholesterol; TAG, triacylglycerol.

**Table 7:** ROC curve for the discriminative ability of the studied hsa-miRs to differentiate HCC from liver cirrhosis either individually or hsa-miRs added to AFP or hsa-miR ratios.

			%		
Variables (Unit)	Cut-Off Point	AUC	Sensitivity	Specificity	*p*-Value *
**AFP (ng/mL)**	>23.3	0.85	69	100	<0.001
**hsa-miR-21-5p-fold changes**	>7.3	0.8	74	45	<0.001
**hsa-miR-155-5p-fold changes**	>1.8	0.7	72	48	<0.01
**hsa-miR-199a-5p fold changes**	<0.44	0.63	79	45	<0.05
**hsa-miR-21-5p-fold changes & AFP**	-	0.888	87	77.5	<0.001
**hsa-miR-155-5p-fold changes & AFP**	-	0.847	82	77.5	<0.001
**hsa-miR-199a-5p fold changes & AFP**	-	0.904	84	80	<0.001
**hsa-miR-21-5p/hsa-miR-199a-5p**	11.45	0.76	95	48	<0.001
**hsa-miR-155-5p/hsa-miR-199a-5p**	2.89	0.71	92	53	<0.01

* Significance at *p* < 0.05 level (2-tailed). AFP, alpha feto protein; AUC, area under the curve.

**Table 8:** Logistic regression analysis using hsa-miR-21-5p, has-miR-155-5p, hsa-miR-192-5p, and hsa-miR199a-5p expression level fold change and their ratios as predictors of HCV-mediated HCC development from liver cirrhosis (n = 79) after adjustment for confounders (age, BMI, RBS, lipids).

			95% C.I.	
Variables (Unit)	*p* Value	OR	Lower	Upper
**hsa-miR-21-5p**	0.002 *	1.189	1.063	1.329
**hsa-miR-155-5p**	NS	0.984	0.881	1.100
**hsa-miR-192-5p**	NS	1.009	0.851	1.196
**hsa-miR-199a-5p**	NS	0.836	0.594	1.177
**hsa-miR-21-5p/hsa-miR-199a-5p**	NS	1.0	1.0	1.0
**hsa-miR-155-5p/hsa-miR-199a-5p**	NS	1.0	0.99	1.0
**Age (years)**	0.027 *	1.132	1.014	1.263
**BMI (Kg/m^2^)**	0.02 *	0.746	0.583	0.955
**TAG/HDL-C**	NS	1.180	0.927	1.501
**TAG**	NS	1.002	0.988	1.016
**TC**	NS	1.004	0.992	1.015
**RBS**	NS	0.993	0.981	1.005

* Significant *p*-value < 0.05. BMI, body mass index; C.I., confidence interval; HCC, hepatocellular carcinoma; HDL, high-density lipoprotein; OR, odds ratio; RBS, random blood sugar; TAG, triacyl glycerol; TC, total cholesterol.

## Data Availability

The original contributions presented in the study are included in the manuscript. Further inquiries can be directed to the corresponding author.

## List of Abbreviations

ADIPQ, adiponectin gene; AFP, alpha-fetoprotein; AGTR1, AGTR1 angiotensin II receptor type 1; Akt, protein kinase B; ALP, alkaline phosphatase; ALT, alanine aminotransferase; AST, aspartate aminotransferase; AUC, area under the curve; BCL2, B-cell lymphoma 2; BCLC, Barcelona Clinic Liver Cancer; BMI, body mass index; C.I., confidence level or interval; CBC, complete blood count; CCL2, chemokine (C-C motif) ligand 2; CD, cluster of differentiation; cDNA, complementary DNA; CHCV, chronic hepatitis C virus; CT, computed tomography; CYR61, cysteine-rich angiogenic inducer 61; D.M., diabetes mellitus; ECLIA, electro-chemiluminescence immunoassay; EDTA, ethylenediaminetetraacetic acid; EGF, epidermal growth factor; ELISA, enzyme-linked immunosorbent test; FC, flow-cytometry; FS, forward scatter; GGT, gamma glutamyl transferase; HBV, hepatitis B virus; HCC, hepatocellular carcinoma; HCV, hepatitis C virus; HDL, high-density lipoprotein; HIF-1, hypoxia-induced factor-1; hsa, homo sapiens; I.C., informed consent; IGF1, insulin-like growth factor 1; IL-6, InterLeukin-6; INR, international normalized ratio; INS, insulin; IR, insulin resistance; KEGG, Kyoto Encyclopedia of Genes and Genomes; KRAS, Kirsten rat sarcoma virus; LC, liver cirrhosis; LN, lymph node; MAP3K4, mitogen-activated protein 3 kinase 4; MAPK8, mitogen-activated protein kinase 8; miRs, microRNAs; mRNA, messenger RNAs; mTOR, mammalian target of rapamycin; nc, non-protein coding; NF-*_k_*B, nuclear factor kappa B cell, NLR, neutrophil-to-lymphocyte ratio; OR, odds ratio; PI3K, phosphatidyl inositol 3kinases; PKB:Akt, protein kinase B; PLR, platelets-to-lymphocytes ratio; PPARG, peroxisome-proliferator-activated receptor gamma; PTEN, phosphatase and tensin homolog tumor suppressor; PV, portal vein; qRT-PCR, quantitative real-time PCR; RBS, random blood sugar; ROC, receiver operating characteristic; SMAD2, suppressor of mothers against decapentaplegic 2; SN, sensitivities; SNORD68, small nucleolar RNA, C/D Box 68; SP, specificities; SPSS, Statistical Package for Social Science software; STAT3, signal transducer and activator of transcription 3; TAG, triacylglycerol; TC, total cholesterol; TGF-B, transforming growth factor beta; TLR4, toll-like receptor 4; TP, tumor protein; UCSC, University of California Santa Cruz; VEGFA, vascular endothelial growth factor A; XBP1, X-box binding protein 1.
